# Synchronous Recurrent Merkel Cell Carcinoma and Primary Cutaneous Melanoma: A Rare Case Report

**DOI:** 10.7759/cureus.90610

**Published:** 2025-08-20

**Authors:** Nektarios Ntalakos, Maria Arnaouti, Evdokia Arkoumani

**Affiliations:** 1 Department of Pathology, Saint Savvas Anticancer Hospital of Athens, Athens, GRC

**Keywords:** dermopathology, primary melanoma, recurrent merkel cell carcinoma, skin disease/dermatology, synchronous occurrence

## Abstract

Merkel cell carcinoma is a rare and aggressive neuroendocrine tumor of the skin, typically affecting elderly patients and arising in sun-damaged areas. It often presents as a painless, firm nodule and has a high recurrence rate. Superficial spreading melanoma is a malignant neoplasm of melanocytes that arises in intermittently sun-exposed areas and progresses through radial and vertical growth phases. While both tumors are individually well-documented, their synchronous occurrence is exceedingly rare. We report a case of a 74-year-old male with a history of Merkel cell carcinoma who presented with two new skin lesions - an ulcerated nodule on the right thigh and a pigmented plaque on the left anterior chest. Histological and immunohistochemical evaluation confirmed the recurrence of Merkel cell carcinoma in the thigh lesion and a primary superficial spreading melanoma in the chest lesion. This case underscores the role of shared pathogenetic factors such as ultraviolet radiation, immunosenescence, and high mutational burden in the development of both malignancies. Diagnostic workup requires comprehensive histopathological and immunohistochemical analysis. Management of both tumors involves wide local excision and sentinel lymph node biopsy, with adjuvant radiotherapy more commonly required in Merkel cell carcinoma due to its high radiosensitivity and immunotherapy playing a central role in advanced disease for both. This case highlights the importance of a multidisciplinary approach for accurate diagnosis and individualized treatment planning.

## Introduction

Merkel cell carcinoma is an uncommon but extremely aggressive neuroendocrine skin tumor, typically arising on chronically sun-exposed areas [1]. Clinically, it often appears as a painless, firm, rapidly growing nodule, though it lacks specific diagnostic features [1]. Regression of the primary lesion is unusual, and recurrence occurs in approximately 40% of cases [1]. In contrast, superficial spreading melanoma is a malignant tumor of melanocytic origin that preferentially develops on areas subjected to intermittent sun exposure [2]. It initially presents as a pigmented, asymmetrical macule that may evolve to display nodularity and ulceration as the disease advances [2].

Although Merkel cell carcinoma and melanoma are individually well-recognized skin malignancies with overlapping etiologic factors such as ultraviolet radiation and immunosuppression, their synchronous presentation is exceedingly rare. This case report describes a 74-year-old male diagnosed with recurrent Merkel cell carcinoma and concurrent superficial spreading melanoma. The case underscores the importance of comprehensive clinicopathological assessment when evaluating patients with multiple suspicious cutaneous lesions.

## Case presentation

A 74-year-old male with a prior history of Merkel cell carcinoma presented to the Dermatology Outpatient Clinic with an ulcerated epidermal lesion on the right thigh. On physical examination, a second pigmented lesion was also noted on the left anterior chest. Both lesions were surgically excised. Due to the patient’s history and clinical findings, a complete lymph node dissection was performed for the thigh lesion.

**Figure 1:**
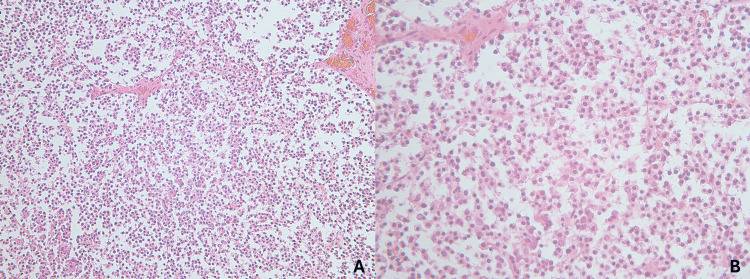
Histopathological imaging of Merkel cell carcinoma. A: H&E stain (X200). B: H&E stain (X400).

**Figure 2:**
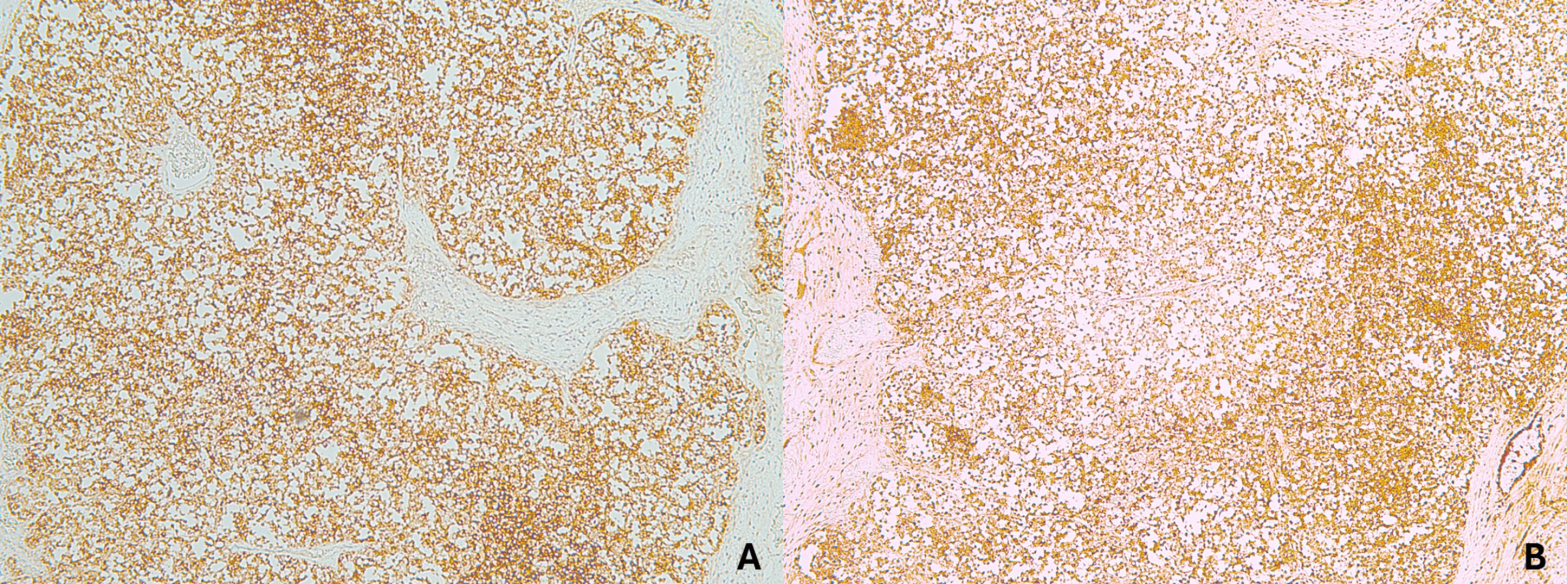
Immunohistochemical analysis of Merkel cell carcinoma. A: Neoplastic cells positive for synaptophysin. B: Neoplastic cells positive for chromogranin.

**Figure 3:**
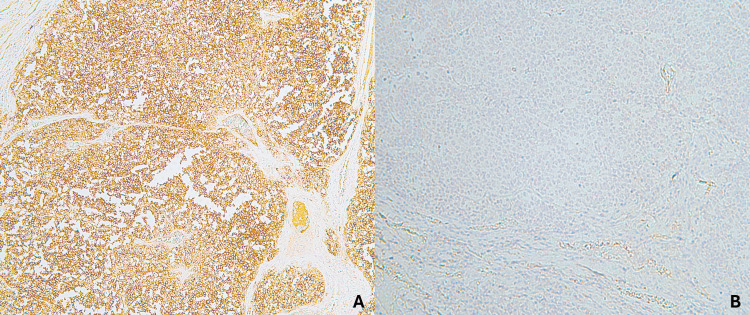
Immunohistochemical analysis of Merkel cell carcinoma. A: Neoplastic cells positive for CD56. B: Neoplastic cells negative for TTF-1.

Gross examination of the right thigh specimen revealed a pale-white, well-circumscribed dermal lesion measuring 55 mm in greatest dimension. The chest lesion consisted of an irregular, blue-black plaque measuring 27 mm. Microscopically, the thigh lesion was a dermal and subcutaneous malignancy composed of diffuse sheets of basophilic tumor cells with vesicular nuclei and small nucleoli (Figure 1). Immunohistochemical staining demonstrated strong positivity for cytokeratin AE1/AE3, CK20, S-100, chromogranin, synaptophysin, and CD56, and negativity for TTF-1, findings consistent with regional recurrence of Merkel cell carcinoma (Figures 2, 3).

Of the four lymph nodes dissected, three showed metastatic involvement by Merkel cell carcinoma. As a result, adjuvant radiotherapy to the nodal basin was administered.

**Figure 4:**
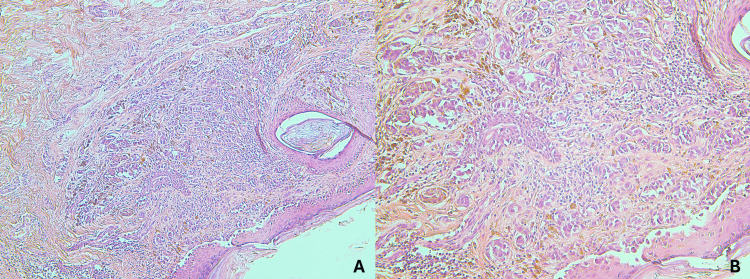
Histopathological imaging of superficial spreading melanoma. A: H&E stain (X100). B: H&E stain (X200).

**Figure 5:**
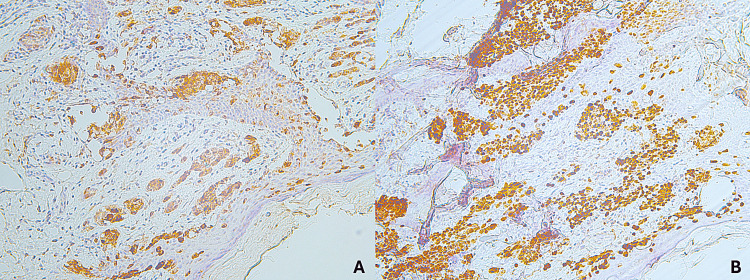
Immunohistochemical analysis of superficial spreading melanoma: A: Neoplastic cells positive for Melan-A. B: Neoplastic cells positive for SOX10.

The second lesion was diagnosed as superficial spreading melanoma, measuring 14 mm in diameter and 0.8 mm in Breslow thickness. Histologically, it showed an asymmetrical proliferation of atypical epithelioid melanocytes with abundant cytoplasm, fine melanin pigmentation, and pleomorphic vesicular nuclei with prominent eosinophilic nucleoli, extending across all layers of the epidermis (Figure 4). Immunohistochemical staining demonstrated strong positivity for Melan-A, HMB-45, and SOX-10 (Figure 5). The lesion exhibited a mitotic rate of 2/mm² without ulceration, corresponding to a pathologic stage of pT1b (Stage IB) according to American Joint Committee on Cancer (AJCC) 8th edition criteria. Due to narrow proximal surgical margins of 4 mm, a wide local re-excision was performed to achieve 2 cm radial margins.

Following review at a multidisciplinary team meeting, the patient was placed under close clinical surveillance. Follow-up included dermatologic skin examinations and whole-body PET-CT scans every six to 12 months for the first three years, with regional lymph node evaluation via ultrasound.

At the three-year follow-up, the patient presented with a new cutaneous lesion on the thorax. Excisional biopsy confirmed the diagnosis of basal cell carcinoma.

## Discussion

Merkel cell carcinoma and cutaneous melanoma are biologically distinct skin malignancies that seldom present concurrently. However, emerging literature indicates that Merkel cell carcinoma may develop alongside other primary cutaneous tumors rather than in isolation [3]. Individuals diagnosed with Merkel cell carcinoma exhibit a heightened risk of secondary skin cancers, possibly due to shared etiological factors such as chronic ultraviolet (UV) radiation exposure, immunosenescence, or immunosuppression [3].

This risk encompasses not only additional primary Merkel cell carcinomas but also an increased incidence of melanoma, squamous cell carcinoma, and basal cell carcinoma [3]. The rare synchronous occurrence of Merkel cell carcinoma and melanoma prompts consideration of overlapping molecular mechanisms. In melanomas arising from sun-protected or intermittently sun-exposed areas, frequently observed genetic alterations include BRAF p.V600E, CDKN2A, TERT promoter, PTEN, and TP53 mutations [2,4]. In contrast, Merkel cell polyomavirus (MCPyV)-negative Merkel cell carcinomas often harbor mutations in TP53 and RB1, while virus-positive tumors exhibit distinct genomic profiles [2,5].

This case highlights the necessity of comprehensive histologic and immunohistochemical analysis, particularly when multiple or atypical skin lesions are present. Precise tumor classification is critical, as therapeutic strategies differ considerably between these malignancies.

Clinically, managing synchronous high-risk cutaneous malignancies introduces complexity in surgical planning, lymph node evaluation, and systemic therapy selection. In Merkel cell carcinoma, treatment is guided primarily by disease stage [5]. For localized and regionally advanced tumors (Stages I-IIIA), wide local excision with histologically negative margins is standard [5]. Sentinel lymph node biopsy is strongly advised for all clinically node-negative patients to detect occult metastases, and baseline imaging with PET/CT is recommended to assess distant disease [5].

In cases with negative lymph node status, surgical excision alone may suffice [5]. However, adjuvant radiotherapy is often employed for patients with high-risk features [5]. For patients with microscopic nodal involvement, complete lymph node dissection may be indicated, with adjuvant nodal radiotherapy considered [5]. Macroscopic nodal disease warrants both complete lymph node dissection and nodal basin irradiation [5]. In advanced or metastatic settings (Stages IIIB-IV), immune checkpoint inhibitors targeting PD-1 or PD-L1 are preferred over traditional chemotherapy [5]. Radiotherapy also remains an effective palliative tool in unresectable or symptomatic cases, with supportive care being recommended for patients who are not candidates for systemic therapy [5]. Surveillance guidelines suggest clinical follow-up every three to six months during the first two to three years and every six to 12 months thereafter up to five years [5].

Melanoma treatment also depends heavily on staging [4]. For in situ disease (Stage 0), wide local excision with 0.5-1.0 cm margins is sufficient [4]. In Stage I disease, T1a tumors require 1 cm margins, while T1b tumors may warrant sentinel lymph node biopsy depending on additional risk factors [4].

Stage II melanomas are typically managed with 1-2 cm excision margins and sentinel lymph node biopsy [4]. For Stage IIC or high-risk tumors, adjuvant therapy with PD-1 inhibitors or, in BRAF-mutated cases, BRAF/MEK inhibitors, may be considered [4]. In Stage III disease, involving regional lymph nodes, wide excision remains central [4]. Positive lymph node biopsy findings may prompt complete lymph node dissection, though observation with imaging is also accepted for micrometastatic disease [4]. Adjuvant immunotherapy or targeted therapy is recommended for one year, and radiotherapy is reserved for select patients with high nodal burden [4].

For Stage IV metastatic melanoma, systemic immunotherapy (PD-1 monotherapy or PD-1/CTLA-4 combination) is first-line [4]. BRAF/MEK inhibition is effective in patients with actionable BRAF mutations [4]. Select patients with limited metastatic burden may benefit from local surgical or radiotherapeutic interventions [4]. Follow-up for melanoma includes clinical assessments every three to six months in the first three years and every six to 12 months thereafter [4]. Imaging is not routinely indicated in early stages unless symptoms arise [4].

In summary, both Merkel cell carcinoma and melanoma require wide excision and sentinel lymph node biopsy for accurate staging [4,5]. However, Merkel cell carcinoma is more likely to require adjuvant radiotherapy due to its radiosensitivity and higher locoregional recurrence risk [5], while melanoma protocols prioritize immunotherapy and targeted therapy based on mutation status [4]. In advanced disease, immune checkpoint inhibitors have significantly improved outcomes in both tumor types [4,5].

Given the absence of standardized guidelines for synchronous Merkel cell carcinoma and melanoma, individualized care guided by a multidisciplinary team, including dermatology, surgical oncology, pathology, and medical oncology, is essential to optimize diagnosis and treatment planning.

## Conclusions

The synchronous occurrence of Merkel cell carcinoma and superficial spreading melanoma is a rare and diagnostically challenging event. Shared pathogenetic mechanisms such as ultraviolet radiation exposure, immunosuppression, and high tumor mutational burden may underlie this coexistence. Comprehensive histopathologic and immunohistochemical evaluation is critical for accurate diagnosis, while a multidisciplinary team is essential for optimizing treatment and improving patient outcomes.
